# Relative thoracic changes from supine to upright patient position: A proton collaborative group study

**DOI:** 10.1002/acm2.14129

**Published:** 2023-08-26

**Authors:** Joseph Marano, Michael W. Kissick, Tracy S. A. Underwood, Steven J. Laub, Michelle Lis, Andries N. Schreuder, Brad Kreydick, Mark Pankuch

**Affiliations:** ^1^ Department of Medical Physics Northwestern Medicine Proton Center Warrenville Illinois USA; ^2^ Leo Cancer Care Middleton Wisconsin USA; ^3^ Department of Medical Physics and Biomedical Engineering Leo Cancer Care Horley UK

**Keywords:** patient positioning, proton therapy, seated treatment

## Abstract

This study presents position changes of a few radiotherapy‐relevant thoracic organs between upright and typical supine patient orientations. Using tools in a commercial treatment planning system (TPS), key anatomical distances were measured for four‐dimensional CT data sets and analyzed for the two patient orientations. The uncertainty was calculated as the 95% confidence interval (CI) on the relative difference for each of the four analyzed changes for upright relative to supine, as follows: the distance of the bottom of the heart to the top of the sternum, it changed +2.6% or +4 mm (95% CI [+0.30%,+4.9%]); the distance of the center of the C3 vertebra to the backrest, it changed +29% (95% CI [+22%,+36%]); the contoured left and right lungs increased their volumes respectively: +17% (95% CI [+12%,+21%]) for the left, and +9.9% (95% CI [+4.1%,+16%]); and lastly, the distance from the top of the sternum to the top of the liver, but its uncertainty far exceeded the average change by a factor of two. This last result is therefore inconclusive, the others show that with 95% confidence that a change in internal positions is observed for lung volumes and heart position that could be important for upright treatments.

## 1 INTRODUCTION

The goal of this paper is to determine if there are significant changes in a few key organ positions, relative to a change in patient position from supine to seated. The context for this paper is of increasing interest in upright/seated, patient positioning for radiation therapy. Upright treatments were more common in the distant past^1^ when treatment planning was still based on 2D images. Since the invention of the CT scanner in the 1970s, truly 3D treatment planning was possible. However, the addition of the CT scanning step to the treatment process favored that patients remain in the recumbent (typically supine) orientation for treatments. This was due to the longer times for scanning and treating. The scans often took a long time to achieve proper image quality,^2^ and longer radiotherapy treatment times seemingly favored a recumbent position for patient comfort at that stage.^3^

The patient position can affect the quality of treatments, because gravity may bring organs at risk (OAR) closer to the planning treatment volume (PTV) making radiotherapy more challenging, or vice versa. In the process of evaluating the impact of different positioning orientations on treatment efficacy, the first step is to establish what is changing, so that treatment planning can be optimal. The next step is to explore the effect on dose calculations. This paper works towards the first step: considering which key organ positions are changing, by how much, and whether this is significant. This paper adds to a growing body of recent research that observes changes in organ positions from recumbent to upright.^4^, ^5^

Much of the efficacy gain in radiotherapy has occurred from technological advancements that separate the treatment dose being applied to the PTV from the OARs as much as is possible.^6^ It is these relative distances that matter most for an effective plan in the upright position.^7^ There have always been indications for upright scanning and treatments such orthopnea, dyspnea, dysphagia, among others.^3^ In addition to those issues, upright positioning may lead to some advantages for radiotherapy treatments themselves, as there may be increased separation between the tumor and some OARs as well as other positioning advantages.

Studied here are four key positional changes as follows: the distance between the bottom of the heart and the top of the sternum, for a measure of superior‐inferior heart location; the distance between the center of the C3 vertebra and the backrest for a measure of patient posture; the contoured left and right lung volumes, for a measure of lung volume; and lastly, the distance from the top of the sternum to the top of the liver, to measure lung movement excursions. The heart position matters for the risk of Radiation Induced Heart Disease (RIHD).^8^, ^9^, ^10^ The patient's neck position from the couch indicates patient posture, which is important for reproducibility in setup and patient comfort and may also influence internal anatomy. An increase in lung volume would likely result in lower mean lung dose, which may be important when treating the lung near tolerance. Finally, the lung excursion measurement may indicate changes in the physiology of respiratory motion between positions. The methods of measuring the positional changes in the treatment planning system (TPS) are presented next, along with the results summary. The uncertainty was calculated with a standard 95% confidence interval (CI) as described in the next section.

## 2 METHODS

The datasets used for this study consist of 18 pairs of 4DCT scans for the contoured lung volume measurements. 15 pairs of 4DCT scans that had no CT artifacts around the heart or the C3 vertebra were used to establish the heart location, and for the patient posture measurements. 14 pairs of 4DCT scans had no CT artifacts around the liver, and these were used for the lung excursion measurements.  Each pair of images included a 4DCT in both the supine and the upright position.  All image pairs were obtained at the Northwestern Medicine Proton Center (NMPC) in Warrenville, Illinois, USA and all patients received radiotherapy at NMPC and provided permission to use anonymized images. Patients provided written consent through the Proton Collaborative Group (PCG) registry study, and all patient data were anonymized prior to evaluation.

Supine 4DCT image pairs were acquired on a GE Light Speed large bore scanner, with a resolution on the order of 1 mm and geometric accuracy < / = 1 mm. A Phillips Brilliance large bore scanner configured by P‐Cure Ltd (Shilat, Israel) was used to acquire upright 4DCT pairs. The resolution and geometric accuracy of the Phillips scanner are both on the order of 1 mm. In this upright scanner, the patient positioning device is reclined backwards at an angle of 20 degrees from the vertical position. This slightly reclined positioning provides the patient with additional comfort and stability relative to the fully vertical position.

The scans were imported and analyzed using a commercial TPS: RayStation version 9A (RaySearch, Sweden). Measurements were made to quantify the variations in the inferior depth of the heart, posture, lung volume, and diaphragm excursion. The posture change was measured to investigate any potential correlations to the other differences between supine and seated positioning.

The 4DCT image sets were acquired and binned into 10 motion phases. Only the 0%, 30%, 60%, and 90% respiratory phases were used to measure the sternum‐to‐heart distances. These sternum‐to‐heart distances were then averaged to obtain a single value. Two orientations were used to measure the distances, sagittal, and coronal. The sagittal and the coronal values were also averaged to obtain a single distance value for each of upright and supine CT scan of each patient. The data between the upright and supine measurements for each patient was paired, and as above, the relative difference was measured, and its uncertainty calculated as described later in this section.

### 2.1 The heart location landmarks

**FIGURE 1:**
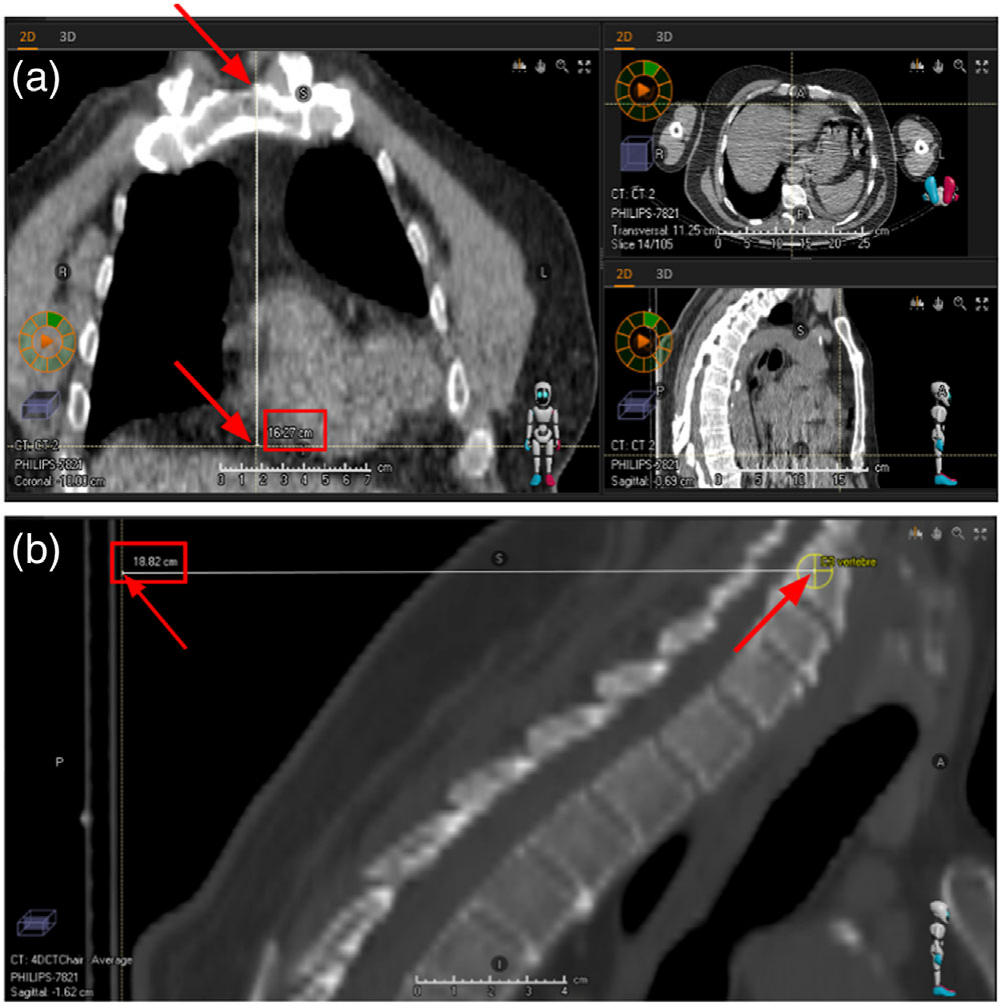
Example measurements, screenshots from the TPS, for patient nine in the upright position. The red boxes indicate the measurement value provided by the software. The red arrows indicate where the thin measurement line ends: (a) heart location measurement example, (b) patient positioning measurement example. TPS, treatment planning system.

The distance measurements for superior/inferior heart location are made from the top of the sternum to the bottom of the heart (Figure 1a). The bottom of the heart was defined as the lowest visible point of the heart. A line was drawn from the top of the sternum to that bottom point, and the length of that line was defined as the distance.

### 2.2 The patient position landmarks

The landmarks used for the patient positioning and posture measurements were the center of the C3 vertebra to the front surface of the backrest (Figure 1b). The distance between these landmarks was measured using both synchronized scrolling and the measurement tool in the TPS.

### 2.3 The lung volume landmarks

The average lung volume measurements were made with contours on the 4DCT average scans (composites of scans from each respiratory phase) for each of the supine and the seated image sets. The average scan was also deformed onto each of the ten phases, along with the lung contours. Maximum and minimum lung volumes for the 10 phases were gathered from each image set and a mid‐point volume was calculated.

### 2.4 The lung excursion landmarks

**FIGURE 2:**
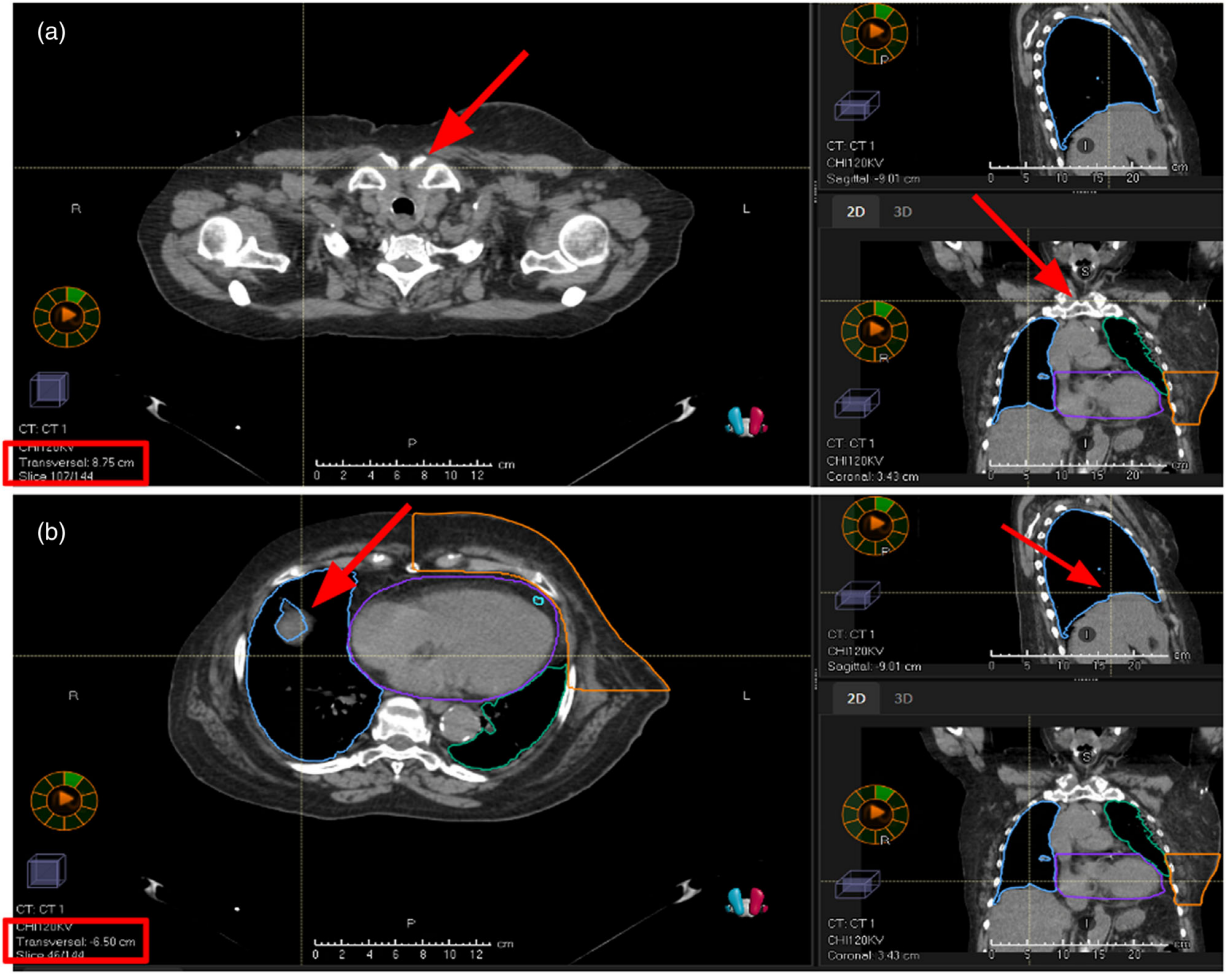
Example measurements, screenshots from the TPS, for patient nine in the supine position.  The red boxes indicate the transversal/axial slice height provided by the software.  The red arrows indicate the landmarks that determine which slices to use: (a) top of sternum slice measurement example, (b) top of liver slice measurement example. TPS, treatment planning system.

The landmarks used for the diaphragm excursion distances are the top of the sternum (Figure 2a) to the top of the liver (i.e., lung/liver interface) (Figure 2b). The top of the liver was defined as the highest visible point of the liver adjacent to the right lung. The axial slice height of both the top of the sternum and the top of the liver were recorded, and the maximum and minimum distance between them throughout the 10 phases was found. These were then subtracted to calculate the amplitude of diaphragm excursion.

### 2.5 Data processing and uncertainty

The average difference in these measurements from supine was calculated: (upright value—supine value)/(supine value). For uncertainty analysis, the sample standard deviation of the relative differences from supine divided by the square root of the sample size, *N*, is first calculated to give the standard error. This standard error is then multiplied by ± 1.96 to provide the uncertainty range for 95% confidence, given a normal distribution that is, indeed satisfied in all cases with a correlation coefficient to normal of greater than 90%.^11^

## 3 RESULTS

The data is highly paired with substantial variation from subject to subject. The uncertainty of the average relative difference is presented here as a CI. In all but the lung excursion measurements, average movements occurred that are on the order of, or exceed, the uncertainty range. The results are summarized in Table 1.

**TABLE 1:** Summary of the average differences and their uncertainty.

Measurement sample size, N	Average relative difference observed	95% Confidence interval of the relative difference average
Heart Location *N* = 15	+2.6%	+/‐ 2.3%
Positioning *N* = 15	+29%	+/‐ 6.6%
Lung Volume (left) *N* = 18	+17%	+/‐ 4.7%
Lung Volume (right) *N* = 18	+9.9%	+/‐ 5.8%
Lung Excursion *N* = 14	+13%	+/‐ 26%

### 3.1 Top of the sternum to the bottom of the heart results

With the 15 subjects in this case, the average change observed was an increase of 2.6%, or 4 mm, upright relative to supine. The uncertainty with a 95% (CI) was found to be in the range [+0.30%, +4.9%] or ± 2.3%.

### 3.2 Positioning: Center of C3 vertebra to the back of the patient positioning device

The horizontal distance between the middle of the C3 vertebra and the front face of the backrest (or couch top in supine positioning) was measured to quantify the variation in patient postures and to correlate with the heart location data (to consider whether neck/spine posture impacted heart location). The same 15 subjects in the heart location cohort are used here. The average change observed was an increase of 29%, upright relative to supine. The uncertainty with a 95% (CI) was found to be in the range [+22%,+36%], or +/‐ 6.6%.

### 3.3 Midpoint lung volume results

**FIGURE 3:**
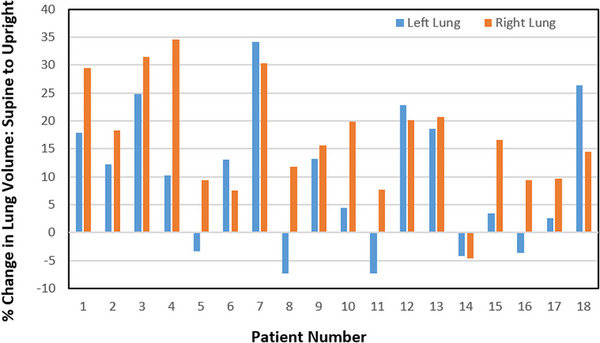
The percentage change in lung volume between the supine and upright position for the left (red bars) and the right (blue bars) lungs respectively.

The volumes of both the left and right lungs were measured to quantify the relative difference from the supine to the upright position (Figure 3). The same 18 subjects were used for both left and right lung measurements. The average relative change observed for the left lung was an increase of 17%, upright relative to supine. The uncertainty with a 95% (CI) was found to be in the range [+12%, +21%], or ± 4.7%. The average relative change observed for the right lung was an increase of 9%, upright relative to supine. The uncertainty with a 95% (CI) was found to be in the range [+4.1%, 16%], or ± 5.8%.

### 3.4 Lung excursion results

**FIGURE 4:**
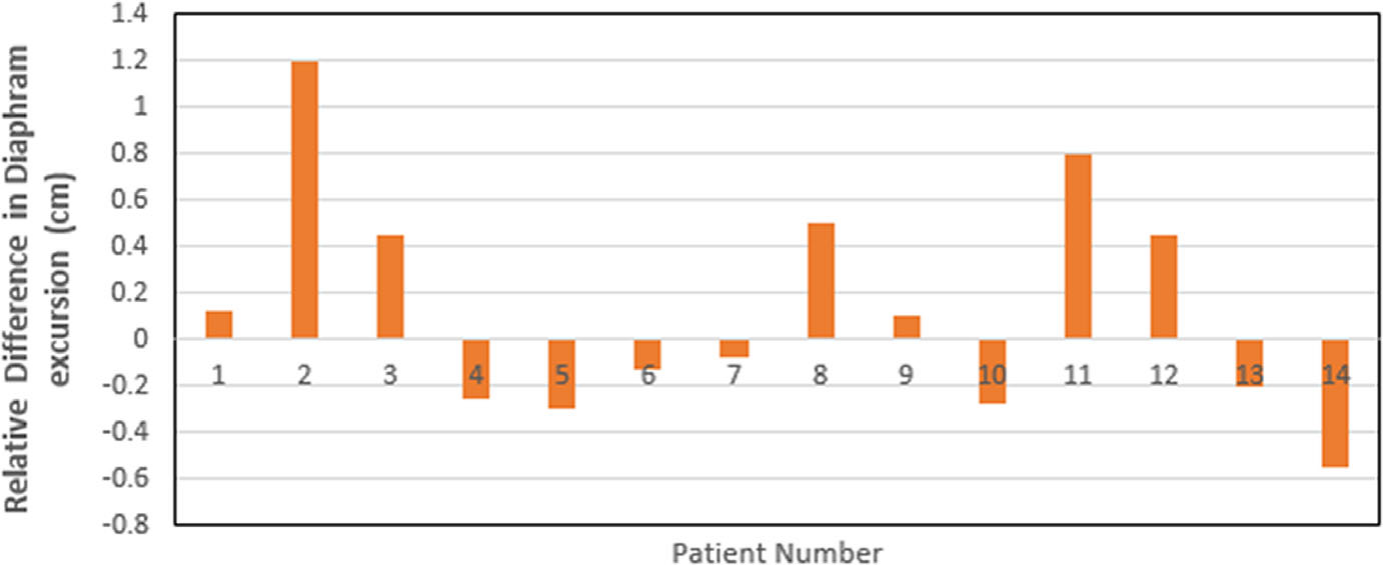
The difference in diaphragm excursion between the upright and supine positions. The change in excursions are relative to the Supine position that is, a positive value means that the excursion is larger in the supine position and vice versa.

The diaphragm movement amplitude was measured to assess the variation in lung excursion between the supine and upright positions. The relative difference in diaphragm excursion between the upright and supine positions for each patient is detailed in Figure 4. The average relative change observed was an increase of 13%, upright relative to supine. The uncertainty with a 95% (CI) was found to be in the range [−13%,+39%], or ± 26%. This result is dominated by uncertainty, and so is considered to be inconclusive.

### 3.5 Discussion and conclusion

The results in this paper indicate that organ position changes do occur in subjects’ anatomies when repositioning from supine to upright. The results presented in Table 1 indicate firstly that there is certainly a difference in positioning of the body from supine to upright with respect to a 95% (CI). The displacement of the heart relative to the body is noticeable relative to the change in positioning, but the variation from subject to subject exceeds the change in the heart position itself. The results also indicate there is an increase in the volumes of both lungs, when comparing upright to supine volumes. However, any change in the lung excursion distances from supine to upright could not be resolved due to the high measurement uncertainty. There are potential benefits from both the heart position change and from the lung volume change that should be investigated in further studies.

When patients lie down on the couch for treatment in supine cases clinically, the liver is observed to deform from the influence of gravity over a period of tens of minutes. The deformation is, on average, several millimeters at its maximum deformation point 12. This situation is similar to our study if one considers that the lung volume changes from gravity are dominated by increased superior‐inferior dimensions, especially near the lower parts of the lungs adjacent to the liver. In our study, the left lung increased its volume by 244 mm^3^ on average, the right lung increased in volume by 197 mm.^3^ Based on these volumetric changes, the change in actual lung position in one direction is on the order of 6 mm. In reality, the superior‐inferior direction will likely dominate this change. The displacements seen in the upright position due to the effects of gravity are on the same order as those previously reported.^12^

The present study's trend towards larger lung volumes for upright patients is consistent with the findings of Yang et al.^4^ and Yamada et al.^13^ As reported by Yang et al., the larger lung volumes associated with upright positioning may allow for a reduction in mean lung dose for patients requiring thoracic radiotherapy.^4^ In this research, diaphragm motion did not significantly vary between upright and supine patient positions, at odds with the finding of Yang et al.,^4^ who observed the magnitude of lung motion to be smaller upright, compared to supine.

Meanwhile, the small but significant increase found in the distance between the top of the sternum and the bottom of the heart (for upright positioning relative to supine) is consistent with an inferior internal organ shift. This agrees with the findings of Reiff et al.,^5^ who observed significant inferior shifts for the kidneys, in going from supine to upright patient positioning. The change in the heart position relative to the sternum could potentially reduce radiation induced cardiovascular disease.^14^

Future work should aim to test/validate the inferior cardiac shift reported here and to consider the position of cardiac substructures such as the left ventricle and the left descending artery^15^ for a larger sample. Treatment planning studies will of course be key to assessing the dosimetric utility of anatomical changes with patient position. Immobilization methods would need to be developed and validated for upright treatments, although recent results on upright comfort and set‐up reproducibility / stability are promising.^16^

## CONFLICT OF INTEREST STATEMENT

This study was partially funded by Leo Cancer Care
